# Investigation of common, low-frequency and rare genome-wide variation in anorexia nervosa

**DOI:** 10.1038/mp.2017.88

**Published:** 2017-07-25

**Authors:** L M Huckins, K Hatzikotoulas, L Southam, L M Thornton, J Steinberg, F Aguilera-McKay, J Treasure, U Schmidt, C Gunasinghe, A Romero, C Curtis, D Rhodes, J Moens, G Kalsi, D Dempster, R Leung, A Keohane, R Burghardt, S Ehrlich, J Hebebrand, A Hinney, A Ludolph, E Walton, P Deloukas, A Hofman, A Palotie, P Palta, F J A van Rooij, K Stirrups, R Adan, C Boni, R Cone, G Dedoussis, E van Furth, F Gonidakis, P Gorwood, J Hudson, J Kaprio, M Kas, A Keski-Rahonen, K Kiezebrink, G-P Knudsen, M C T Slof-Op 't Landt, M Maj, A M Monteleone, P Monteleone, A H Raevuori, T Reichborn-Kjennerud, F Tozzi, A Tsitsika, A van Elburg, Eating Disorder Working Group of the Psychiatric Genomics Consortium, R A H Adan, L Alfredsson, T Ando, O A Andreassen, H Aschauer, J H Baker, J C Barrett, V Bencko, A W Bergen, W H Berrettini, A Birgegard, C Boni, V Boraska Perica, H Brandt, G Breen, C M Bulik, L Carlberg, M Cassina, S Cichon, M Clementi, S Cohen-Woods, J Coleman, R D Cone, P Courtet, S Crawford, S Crow, J Crowley, U N Danner, O S P Davis, M de Zwaan, G Dedoussis, D Degortes, J E DeSocio, D M Dick, D Dikeos, C Dina, B Ding, M Dmitrzak-Weglarz, E Docampo, L Duncan, K Egberts, S Ehrlich, G Escaramís, T Esko, T Espeseth, X Estivill, A Favaro, F Fernández-Aranda, M M Fichter, C Finan, K Fischer, J A B Floyd, L Foretova, M Forzan, C S Franklin, S Gallinger, G Gambaro, H A Gaspar, I Giegling, F Gonidakis, P Gorwood, M Gratacos, S Guillaume, Y Guo, H Hakonarson, K A Halmi, K Hatzikotoulas, J Hauser, J Hebebrand, S Helder, S Herms, B Herpertz-Dahlmann, W Herzog, C E Hilliard, A Hinney, C Hübel, L M Huckins, J I Hudson, J Huemer, H Inoko, V Janout, S Jiménez-Murcia, C Johnson, A Julià, A Juréus, G Kalsi, D Kaminska, A S Kaplan, J Kaprio, L Karhunen, A Karwautz, M J H Kas, W Kaye, J L Kennedy, A Keski-Rahkonen, K Kiezebrink, L Klareskog, K L Klump, G P S Knudsen, B P C Koeleman, D Koubek, M C La Via, M Landén, S Le Hellard, R D Levitan, D Li, P Lichtenstein, L Lilenfeld, J Lissowska, A Lundervold, P Magistretti, M Maj, K Mannik, S Marsal, N Martin, M Mattingsdal, S McDevitt, P McGuffin, E Merl, A Metspalu, I Meulenbelt, N Micali, J Mitchell, K Mitchell, P Monteleone, A M Monteleone, P Mortensen, M A Munn-Chernoff, M Navratilova, I Nilsson, C Norring, I Ntalla, R A Ophoff, J K O'Toole, A Palotie, J Pante, H Papezova, D Pinto, R Rabionet, A Raevuori, A Rajewski, N Ramoz, N W Rayner, T Reichborn-Kjennerud, S Ripatti, M Roberts, A Rotondo, D Rujescu, F Rybakowski, P Santonastaso, A Scherag, S W Scherer, U Schmidt, N J Schork, A Schosser, L Slachtova, R Sladek, P E Slagboom, M C T Slof-Op 't Landt, A Slopien, N Soranzo, L Southam, V M Steen, E Strengman, M Strober, P F Sullivan, J P Szatkiewicz, N Szeszenia-Dabrowska, I Tachmazidou, E Tenconi, L M Thornton, A Tortorella, F Tozzi, J Treasure, A Tsitsika, K Tziouvas, A A van Elburg, E F van Furth, G Wagner, E Walton, H Watson, H-E Wichmann, E Widen, D B Woodside, J Yanovski, S Yao, Z Yilmaz, E Zeggini, S Zerwas, S Zipfel, D A Collier, P F Sullivan, G Breen, C M Bulik, E Zeggini

**Affiliations:** 1 Department of Human Genetics, Wellcome Trust Sanger Institute, Wellcome Trust Genome Campus, Hinxton, Cambridge, UK; 2 Division of Psychiatric Genomics, Icahn School of Medicine at Mount Sinai, New York, NY, USA; 3 Department of Psychiatry and Nutrition, University of North Carolina at Chapel Hill, Chapel Hill, NC, USA; 4 Section of Eating Disorders, Institute of Psychiatry, Psychology and Neuroscience, King's College London, London, UK; 5 NIHR BRC SLaM BioResource for Mental Health, SGDP Centre & Centre for Neuroimaging Sciences, Section of Eating Disorders, Institute of Psychiatry, Psychology and Neuroscience, King's College London, London, UK; 6 Klinik für Kinder- und Jugendpsychiatrie, Psychotherapie und Psychosomatik Klinikum Frankfurt, Frankfurt, Germany; 7 Division of Psychological and Social Medicine and Developmental Neurosciences, Faculty of Medicine, TU Dresden, Dresden, Germany; 8 Eating Disorders Research and Treatment Center, Department of Child and Adolescent Psychiatry, Faculty of Medicine, TU Dresden, Dresden, Germany; 9 Department of Child and Adolescent Psychiatry and Psychotherapy, University Hospital Essen, University of Duisburg-Essen, Essen, Germany; 10 Helmholtz Zentrum München, Deutsches Forschungszentrum für Gesundheit und Umwelt, Neuherberg, Germany; 11 Division of Psychological & Social Medicine and Developmental Neurosciences, Technische Universität Dresden, Faculty of Medicine, University Hospital C.G. Carus, Dresden, Germany; 12 Department of Psychology, Georgia State University, Atlanta, GA, USA; 13 Erasmus University Medical Center, Rotterdam, The Netherlands; 14 Center for Human Genome Research at the Massachusetts General Hospital, Boston, MA, USA; 15 Department of Public Health & Institute for Molecular Medicine FIMM, University of Helsinki, Helsinki, Finland; 16 Brain Center Rudolf Magnus, Department of Neuroscience and Pharmacology, University Medical Center Utrecht, Utrecht, The Netherlands; 17 INSERM U984, Centre of Psychiatry and Neuroscience, Paris, France; 18 Mary Sue Coleman Director, Life Sciences Institute, Professor of Molecular and Integrative Physiology, University of Michigan, Ann Arbor, MI, USA; 19 Department of Dietetics-Nutrition, Harokopio University, Athens, Greece; 20 Rivierduinen Eating Disorders Ursula, Leiden, Zuid-Holland, The Netherlands; 21 Eating Disorders Unit, 1st Department of Psychiatry, National and Kapodistrian University of Athens, Medical School, Athens, Greece; 22 Department of Psychiatry, McLean Hospital/Harvard Medical School, Belmont, MA, USA; 23 Groningen Institute for Evolutionary Life Sciences, University of Groningen, Groningen, The Netherlands; 24 Department of Public Health, Clinicum, University of Helsinki, Helsinki, Finland; 25 Institute of Applied Health Sciences, University of Aberdeen, Aberdeen, UK; 26 Health Data and Digitalisation, Norwegian Institute of Public Health, Oslo, Norway; 27 Department of Psychiatry, University of Naples SUN, Naples, Italy; 28 Department of Medicine and Surgery, Section of Neurosciences, University of Salerno, Salerno, Italy; 29 Department of Genetics, Environment and Mental Health, Norwegian Institute of Public Health, Oslo, Norway; 30 eHealth Lab-Computer Science Department, University of Cyprus, Nicosia, Cyprus; 31 Adolescent Health Unit (A.H.U.), 2nd Department of Pediatrics – Medical School, University of Athens "P. & A. Kyriakou" Children's Hospital, Athens, Greece; 32 Center for Eating Disorders Rintveld, University of Utrecht, Utrecht, The Netherlands; 33 Eli Lilly and Company, Erl Wood Manor, Windlesham, UK; 34 Departments of Genetics and Psychiatry, University of North Carolina at Chapel Hill, Chapel Hill, NC, USA; 35 Department of Medical Epidemiology and Biostatistics, Karolinksa Institutet, Stockholm, Sweden; 36 Social Genetic and Developmental Psychiatry, King's College London, London, UK; 37 University Medical Center Utrecht, Utrecht, The Netherlands; 38 Altrecht Eating Disorders Rintveld, Zeist, The Netherlands; 39 Institute of Environmental Medicine, Karolinska Institute, Stockholm, Sweden; 40 Department of Psychosomatic Research, National Institute of Mental Health, National Center of Neurology and Psychiatry, Tokyo, Japan; 41 Division of Mental Health and Addiction, NORMENT, K.G. Jebsen Centre for Psychosis Research, Oslo University Hospital, Oslo, Norway; 42 Institute of Clinical Medicine, University of Oslo, Oslo, Norway; 43 Biopsychosocial Corporation, Vienna, Austria; 44 Department of Psychiatry, University of North Carolina at Chapel Hill, Chapel Hill, NC, USA; 45 Wellcome Trust Sanger Institute, Wellcome Trust Genome Campus, Hinxton, UK; 46 Institute of Hygiene and Epidemiology, First Faculty of Medicine, Charles University, Prague, Czech Republic; 47 Biorealm Research, Culver City, CA, USA; 48 Perelman School of Medicine at the University of Pennsylvania, Philadelphia, PA, USA; 49 Department of Clinical Neuroscience, Karolinska Institutet, Stockholm, Sweden; 50 Stockholm Health Care Services, Stockholm County Council, Stockholm, Sweden; 51 INSERM U984, Centre of Psychiatry and Neuroscience, Paris, France; 52 University of Split School of Medicine, Split, Croatia; 53 The Center for Eating Disorders at Sheppard Pratt, Baltimore, MD, USA; 54 Social Genetic and Developmental Psychiatry, King's College London, London, UK; 55 NIHR BRC for Mental Health, Institute of Psychiatry, Psychology and Neuroscience, King's College London, SLaM NHS Trust, London, UK; 56 Department of Psychiatry and Nutrition, University of North Carolina at Chapel Hill, Chapel Hill, NC, USA; 57 Karolinska Institutet, Stockholm, Sweden; 58 Department of Psychiatry, Medical University of Vienna, Vienna, Austria; 59 Clinical Genetics Unit, Department of Women’s and Children’s Health, University of Padova, Padova, Italy; 60 Department of Biomedicine, University Hospital Basel, Basel, Switzerland; 61 Department of Genomics, Life and Brain Center, Institute of Human Genetics, University of Bonn, Bonn, Germany; 62 Institute of Neuroscience and Medicine, Research Center Jülich, Jülich, Germany; 63 School of Psychology, Finders University, Adelaide, SA, Australia; 64 Medical Research Council Social Genetic and Developmental Psychiatry Centre, Institute of Psychiatry, King's College London, London, UK; 65 Social, Genetic and Developmental Psychiatry Centre, Institute of Psychiatry, King's College London, London, UK; 66 Department of Molecular Physiology and Biophysics, Vanderbilt University School of Medicine, Nashville, TN, USA; 67 CHRU Montpellier, University of Montpellier, Montpellier, France; 68 Department of Psychiatry, University of Minnesota, Minneapolis, MN, USA; 69 Department of Genetics, University of North Carolina at Chapel Hill, Chapel Hill, NC, USA; 70 MRC Integrative Epidemiology Unit, University of Bristol, Bristol, UK; 71 Department of Psychosomatic Medicine and Psychotherapy, Hannover Medical School, Hannover, Germany; 72 Department of Dietetics-Nutrition, Harokopio University, Athens, Greece; 73 Department of Neurosciences, University of Padova, Padova, Italy; 74 Seattle University College of Nursing, Seattle, WA, USA; 75 Department of Psychiatry, Virginia Institute for Psychiatric and Behavioral Genetics, Virginia Commonwealth University, Richmond, VA, USA; 76 First Department of Psychiatry, Athens University Medical School, Athens, Greece; 77 University of Nantes, Nantes, France; 78 Department of Chemistry and Biochemistry, University of California at San Diego, La Jolla, CA, USA; 79 Laboratory of Psychiatric Genetics, Poznan University of Medical Sciences, Poznan, Poland; 80 The Barcelona Institute of Science and Technology, Universitat Pompeu Fabra, CIBER Epidemiología y Salud Pública Barcelona, Barcelona, Spain; 81 Interdisciplinary Cluster for Applied Genoproteomics-Université de Liège, Liège, Belgium; 82 Stanford University, Stanford, CA, USA; 83 Broad Institute of MIT and Harvard, Massachusetts General Hospital, MA, USA; 84 Department of Child and Adolescent Psychiatry, Psychosomatics and Psychotherapy, University Hospital of Würzburg, Würzburg, Germany; 85 Division of Psychological and Social Medicine and Developmental Neurosciences, Faculty of Medicine, TU Dresden, Germany; 86 Eating Disorders Research and Treatment Center, Department of Child and Adolescent Psychiatry, Faculty of Medicine, TU Dresden, Germany; 87 The Barcelona Institute of Science and Technology, Universitat Pompeu Fabra, Centro de Investigación Biomédica en Red en Epidemiología y Salud Pública Barcelona, Barcelona, Spain; 88 Estonian Genome Center, University of Tartu, Tartu, Estonia; 89 Medical and Population Genetics Program, Broad Institute of MIT and Harvard, Cambridge, MA, USA; 90 Division of Endocrinology, Boston Children's Hospital, Boston, MA, USA; 91 Department of Psychology, University of Oslo, Oslo, Norway; 92 Centre for Genomic Regulation, The Barcelona Institute of Science and Technology, Universitat Pompeu Fabra, Centro de Investigación Biomédica en Red en Epidemiología y Salud Pública Barcelona, Barcelona, Spain; 93 University Hospital of Bellvitge-IDIBELL, University of Barcelona, Barcelona, Spain; 94 Centro de Investigación Biomédica en Red CIBEROBN, Instituto de Salud Carlos III, Madrid, Spain; 95 Ludwig-Maximilians-University Munich, Schön Klinik Roseneck, Germany; 96 UCL Institute of Cardiovascular Sciences, London, UK; 97 Department of Cancer Epidemiology and Genetics, Masaryk Memorial Cancer Institute, Brno, Czech Republic; 98 Clinical Genetics Unit, University Hospital of Padova, Padova, Italy; 99 Lunenfeld-Tanenbaum Research Institute of Mount Sinai Hospital, University of Toronto, Toronto, ON, Canada; 100 Ontario Institute for Cancer Research, Toronto, ON, Canada; 101 Division of Nephrology and Dialysis Columbus-Gemelli University Hospital, Rome, Italy; 102 Martin Luther University of Halle- Wittenberg, Halle, Germany; 103 Eating Disorders Unit, First Department of Psychiatry, National and Kapodistrian University of Athens, Medical School, Athens, Greece; 104 INSERM U984, Sainte- Anne Hospital, University of Paris-Descartes, Paris, France; 105 Center for Applied Genomics, Children's Hospital of Philadelphia, Philadelphia, PA, USA; 106 Children's Hospital of Philadelphia, University of Pennsylvania, Philadelphia, PA, USA; 107 The Division of Human Genetics, Department of Pediatrics, Perelman School of Medicine, Philadelphia, PA, USA; 108 Department of Psychiatry, Weill Cornell Medical College, New York, NY, USA; 109 Psychiatric Genetics Unit, Department of Psychiatry, Poznan University of Medical Sciences, Poznan, Poland; 110 Department of Child and Adolescent Psychiatry and Psychotherapy, University Hospital Essen, University of Duisburg-Essen, Essen, Germany; 111; 112 Zorg op Orde, Leidschendam, The Netherlands; 113 King’s College London, London, UK; 114 Division of Medical Genetics, Department of Biomedicine, University of Basel, Basel, Switzerland; 115 Department of Child and Adolescent Psychiatry, Psychosomatics and Psychotherapy of the RWTH Aachen, Aachen, Germany; 116 Department of Psychosocial and Internal Medicine, Heidelberg University, Heidelberg, Germany; 117 Lineberger Comprehensive Cancer Center, The University of North Carolina at Chapel Hill, Chapel Hill, NC, USA; 118 Social, Genetic and Developmental Psychiatry Centre, Institute of Psychiatry, Psychology and Neuroscience, King's College London, London, UK; 119 Icahn School of Medicine, Mount Sinai, NY, USA; 120 Department of Psychiatry, McLean Hospital, Harvard Medical School, Belmont, MA, USA; 121 Eating Disorders Unit, Department of Child and Adolescent Psychiatry, Medical University of Vienna, Vienna, Austria; 122 Department of Molecular Life Sciences, Tokai University School of Medicine, Kanagawa, Japan; 123 Department of Epidemiology and Public Health, Ostrava University, Ostrava, Czech Republic; 124 Department of Psychiatry and CIBEROBN, University Hospital of Bellvitge-IDIBELL, University of Barcelona, Barcelona, Spain; 125 Department of Clinical Sciences, School of Medicine, University of Barcelona, Barcelona, Spain; 126 Eating Recovery Center, Denver, CO, USA; 127 Rheumatology Research Group, Vall d’Hebron Hospital Research Institute, Barcelona, Spain; 128 Department of Medical Epidemiology and Biostatistics, Karolinska Institutet, Stockholm, Sweden; 129 Social, Genetic and Developmental Psychology, King's College London, London, UK; 130 Department of Psychiatry, First Faculty of Medicine, Charles University, Prague, Czech Republic; 131 Department of Psychiatry, Center for Addiction and Mental Health, Institute of Medical Science, University of Toronto, Toronto, Canada; 132 University of Helsinki, National Institute for Health and Welfare, Helsinki, Finland; 133 Institute for Molecular Medicine Finland FIMM, University of Helsinki, Helsinki, Finland; 134 Institute of Public Health and Clinical Nutrition, University of Eastern Finland, Kuopio, Finland; 135 Groningen Institute for Evolutionary Life Sciences, University of Groningen, Groningen, The Netherlands; 136 Brain Center Rudolf Magnus, Department of Translational Neuroscience, University Medical Center Utrecht, Utrecht, The Netherlands; 137 Department of Psychiatry, University of California, San Diego, CA, USA; 138 Center for Addiction and Mental Health, University of Toronto, Toronto, ON, Canada; 139 Department of Public Health, Clinicum, University of Helsinki, Helsinki, Finland; 140 Institute of Applied Health Sciences, University of Aberdeen, Aberdeen, UK; 141 Department of Medicine, Unit of Rheumatology, Karolinska University Hospital, Stockholm, Sweden; 142 Department of Psychology, Michigan State University, East Lansing, MI, USA; 143 Health Data and Digitalisation, Norwegian Institute of Public Health, Oslo, Norway; 144 Department of Medical Genetics, University Medical Center Utrecht, Utrecht, The Netherlands; 145 Gothenburg University, Gothenburg, Sweden; 146 NORMENT— K.G. Jebsen Center for Psychosis Research, Department of Clinical Science, University of Bergen, Bergen, Norway; 147 Dr Einar Martens Research Group for Biological Psychiatry, Center for Medical Genetics and Molecular Medicine, Haukeland University Hospital, Bergen, Norway; 148 Department of Psychiatry, University of Toronto, Toronto, ON, Canada; 149 Department of Clinical Psychology, American School of Professional Psychology at Argosy University Washington DC, Washington, DC, USA; 150 M. Sklodowska-Curie Cancer Center and Institute of Oncology, Warsaw, Poland; 151 Department of Biological and Medical Psychology, University of Bergen, Bergen, Norway; 152 K. G. Jebsen Center for Neuropsychiatric Disorders Norway, Oslo, Norway; 153 Brain Mind Institute, EPFL, Center for Psychiatric Neuroscience, Department of Psychiatry—CHUV/UNIL, Lausanne, Switzerland; 154 Division of Biological and Environmental Sciences and Engineering, King Abdullah University of Science and Technology, Thuwal, Saudi Arabia; 155 Department of Psychiatry, University of Naples SUN, Naples, Italy; 156 Institute of Molecular and Cell Biology, University of Tartu, Tartu, Estonia; 157 Center for Integrative Genomics, University of Lausanne, Lausanne, Switzerland; 158 QIMR Berghofer Medical Research Institute, Brisbane, QLD, Australia; 159 NORMENT, University of Oslo, Oslo, Norway; 160 Division of Mental Health and Addiction, Oslo University Hospital, Oslo, Norway; 161 University College Cork, Health Service Executive South, Cork, Ireland; 162 MRC Social Genetic and Developmental Psychiatry Centre, Kings College London, London, UK; 163 Leiden University Medical Centre, Molecular Epidemiology Section (Department of Medical Statistics), Leiden, The Netherlands; 164 Department of Psychiatry, Icahn School of Medicine at Mount Sinai, New York, NY, USA; 165 Department of Psychiatry and Behavioral Science, University of North Dakota School of Medicine and Health Sciences, Fargo, ND, USA; 166 National Center for PTSD, VA Boston Healthcare System, Boston, MA, USA; 167 Boston University School of Medicine, Boston, MA, USA; 168 Department of Medicine and Surgery, Section of Neurosciences, University of Salerno, Salerno, Italy; 169 University of Naples SUN, Naples, Italy; 170 Aarhus University, Denmark; 171 Department of Molecular Medicine and Surgery, Karolinska Institutet, Stockholm, Sweden; 172 Center for Molecular Medicine, Karolinska University Hospital, Stockholm, Sweden; 173 Harakopio University, Athens, Greece; 174 Center for Neurobehavioral Genetics, Semel Institute for Neuroscience and Human Behavior, University of California Los Angeles, Los Angeles, CA, USA; 175 Kartini Clinic, Portland, OR, USA; 176 Institute for Molecular Medicine Finland, Helsinki, Finland; 177 Centre de Psychiatrie et Neurosciences— Inserm U894, Paris, France; 178 Department of Psychiatry, First Faculty of Medicine, Charles University, Prague, Czech Republic; 179 Department of Psychiatry, Department of Genetics and Genomic Sciences, Icahn School of Medicine at Mount Sinai, Mount Sinai, NY, USA; 180 University of Helsinki, Helsinki University Central Hospital, Helsinki, Finland; 181 University of Turku, Turku, Finland; 182 Department of Child and Adolescent Psychiatry, Poznan University of Medical Sciences, Poznan, Poland; 183 Wellcome Trust Centre for Human Genetics, University of Oxford, Oxford, UK; 184 Oxford Centre for Diabetes, Endocrinology and Metabolism, University of Oxford, Oxford, UK; 185 Department of Genetics, Environment and Mental Health, Norwegian Institute of Public Health, University of Oslo, Oslo, Norway; 186 Department of Biometry, University of Helsinki, Helsinki, Finland; 187 Section of Eating Disorders, Institute of Psychiatry, Psychology and Neuroscience, King's College London, London, UK; 188 University of Pisa, Pisa, Italy; 189 Ludwig-Maximilians-University Munich, Munich, Germany; 190 Institute of Psychiatry and Neurology, University of Social Sciences and Humanities, Warsaw, Poland; 191 Research Group Clinical Epidemiology, Center for Sepsis Control and Care, Jena University Hospital, Jena, Germany; 192 The Centre for Applied Genomics, Genetics and Genome Biology, The Hospital for Sick Children, Toronto, ON, Canada; 193 McLaughlin Centre and Department of Molecular Genetics, University of Toronto, Toronto, ON, USA; 194 Department of Human Biology, J. Craig Venter Institute, La Jolla, CA, USA; 195 Department of Psychiatry and Psychotherapy, Medical University of Vienna, Vienna, Austria; 196 Department of Pediatrics and Center of Applied Genomics, First Faculty of Medicine, Charles University, Prague, Czech Republic; 197 McGill University and Génome Québec Innovation Centre, Montreal, QC, Canada; 198 Leiden University Medical Centre, Leiden, The Netherlands; 199 Rivierduinen Eating Disorders Ursula, Leiden University Medical Centre, Leiden, The Netherlands; 200 Department of Psychiatry, Poznan University of Medical Sciences, Poznan, Poland; 201 Department of Human Genetics, Wellcome Trust Sanger Institute, Wellcome Trust Genome Campus, Hinxton, UK; 202 Department of Haematology, University of Cambridge, Hills Rd, Cambridge CB2 0AH, UK; 203 The National Institute for Health Research Blood and Transplant Unit (NIHR BTRU) in Donor Health and Genomics at the University of Cambridge, Cambridge, UK; 204 Department of Clinical Science, K.G. Jebsen Centre for Psychosis Research, Norwegian Centre For Mental Disorders Research, University of Bergen, Bergen, Norway; 205 Dr Einar Martens Research Group for Biological Psychiatry, Center for Medical Genetics and Molecular Medicine, Haukeland University Hospital, Bergen, Norway; 206 Department of Pathology, University Medical Center Utrecht, Utrecht, The Netherlands; 207 Department of Psychiatry and Biobehavioral Sciences, University of California Los Angeles, Los Angeles, CA, USA; 208 David Geffen School of Medicine, University of California, Los Angeles, CA, USA; 209 Department of Genetics and Psychiatry, University of North Carolina at Chapel Hill, Chapel Hill, NC, USA; 210 Nofer Institute of Occupational Medicine, Department of Environmental Epidemiology, Lodz, Poland; 211 University of Padova, Padova, Italy; 212 University of Perugia, Perugia, Italy; 213 eHealth Lab-Computer Science Department, University of Cyprus, Nicosia, Cyprus; 214 Adolescent Health Unit (A.H.U.), 2nd Department of Pediatrics—Medical School, University of Athens “P. & A. Kyriakou” Children's Hospital, Athens, Greece; 215 “P. & A. Kyriakou” Children's Hospital, Athens, Greece; 216 Center for Eating Disorders Rintveld, Altrecht, The Netherlands; 217 Rivierduinen Eating Disorders Ursula, Leiden, The Netherlands; 218 Department of Psychiatry, Leiden University Medical Center, Leiden, The Netherlands; 219 Department of Psychology, Georgia State University, Atlanta, GA, USA; 220 Department of Psychology, Institute of Psychiatry, Psychology and Neuroscience, King's College London, London, UK; 221 University of North Carolina at Chapel Hill, School of Paediatrics and Child Health, Faculty of Medicine, Dentistry, and Life Sciences, The University of Western Australia, Perth, WA, Australia; 222 School of Psychology and Speech Pathology, Faculty of Health Sciences, Curtin University, Perth, WA, Australia; 223 Helmholtz Centre Munich—German Research Center for Environmental Health, Munich, Germany; 224 University of Toronto, Toronto, ON, Canada; 225 Inpatient Eating Disorders Service, Toronto General Hospital, Toronto, ON, Canada; 226 Section on Growth and Obesity, Program in Developmental Endocrinology and Genetics, Eunice Kennedy Shriver National Institute of Child Health and Human Development, National Institutes of Health, Bethesda, MD, USA; 227 Department of Internal Medicine VI, Psychosomatic Medicine and Psychotherapy, University Medical Hospital Tuebingen, Tuebingen, Germany

## Abstract

Anorexia nervosa (AN) is a complex neuropsychiatric disorder presenting with dangerously low body weight, and a deep and persistent fear of gaining weight. To date, only one genome-wide significant locus associated with AN has been identified. We performed an exome-chip based genome-wide association studies (GWAS) in 2158 cases from nine populations of European origin and 15 485 ancestrally matched controls. Unlike previous studies, this GWAS also probed association in low-frequency and rare variants. Sixteen independent variants were taken forward for *in silico* and *de novo* replication (11 common and 5 rare). No findings reached genome-wide significance. Two notable common variants were identified: rs10791286, an intronic variant in *OPCML* (*P*=9.89 × 10^−6^), and rs7700147, an intergenic variant (*P*=2.93 × 10^−5^). No low-frequency variant associations were identified at genome-wide significance, although the study was well-powered to detect low-frequency variants with large effect sizes, suggesting that there may be no AN loci in this genomic search space with large effect sizes.

## Introduction

Family studies of anorexia nervosa (AN) have consistently shown that first-degree relatives of AN sufferers have an increased risk of AN, compared with relatives of unaffected individuals.^1, 2, 3, 4^ Twin studies have estimated the heritability of AN at 56%,^5^ with the majority of remaining variance in liability attributed to non-shared environmental factors (38%).^5^

Three genome-wide association studies (GWAS) of AN have been conducted to date. The first comprised 1033 AN cases collected as part of the Price Foundation Genetic Study of Anorexia Nervosa and 3733 pediatric controls from the Children’s Hospital of Philadelphia.^6^ This study focused on common variation and identified 11 suggestive variants (*P*<1 × 10^−5^). None reached genome-wide significance in the primary analysis, although one variant (rs4479806) approached genome-wide significance in an associated secondary analysis. The second study (comprising 2907 cases and 14 860 controls) was carried out by the Genetic Consortium for AN, as part of the Wellcome Trust Case Control Consortium 3 (WTCCC3) effort.^7^ This study identified two suggestively associated variants (*P*<1 × 10^−5^). Notably, signals at *P*<1 × 10^−5^ were significantly more likely to have the same direction of effect in the replication as in the discovery cohorts (*P*=4 × 10^−6^), which implies that true signals exist within this data set, but that the study was underpowered for detection. Recently, a third study-meta-analyzed samples from both of these studies, as well as some novel cases, comprising a total of 3495 cases and 10 982 controls. To our knowledge, this study identified the first genome-wide significant locus for AN (index variant rs4622308, *P*=4.3 × 10^−9^).^8^

Both previous studies focused on common variation. Here, we conducted, to our knowledge, the first association study that also considered low frequency (minor allele frequency (MAF)<5%) and rare exonic variants in addition to common variation.

## Materials and methods

### Sample collections

**Figure 1:**
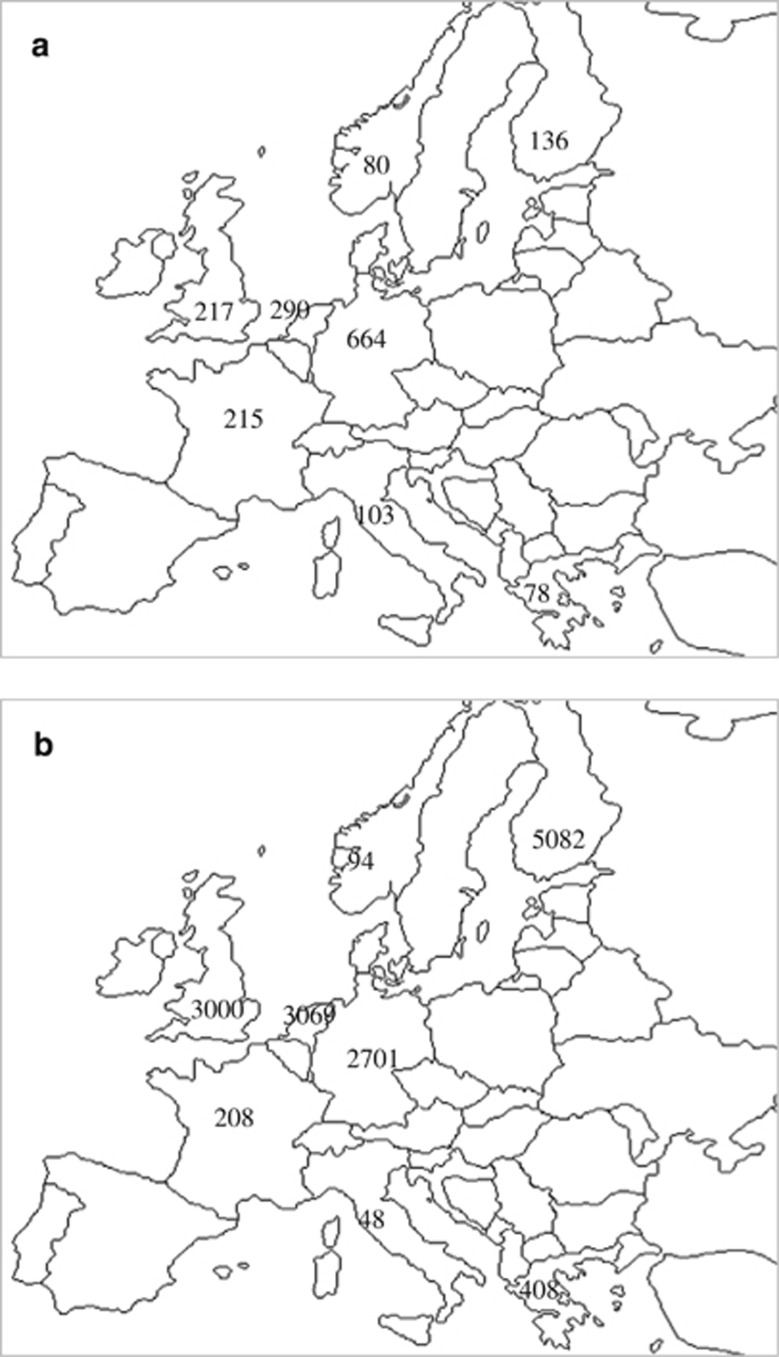
Geographical distribution of samples across Europe. (**a**) Distribution of cases across Europe; 375 USA cases are not shown in this diagram. (**b**) Distribution of controls across Europe; 873 USA controls are not shown in this diagram.

We conducted a GWAS across nine discovery data sets (the majority overlapping with Genetic Consortium for AN, as part of the Wellcome Trust Case Control Consortium 3 (WTCCC3/WTCCC3 samples), resulting in a total of 2158 cases and 15 485 ancestrally matched controls (Table 1 and Figure 1). All AN cases were female. AN diagnosis was made via semistructured or structured interview, or population assessment strategy using Diagnostic and Statistical Manual of Mental Disorders (DSM)-IV criteria for AN. The amenorrhea criterion was not applied, as this has been shown not to be diagnostically relevant^9^ and has since been dropped from DSM-5.^10, 11^ All cases met criteria for lifetime AN.

Exclusion criteria included confounding medical diagnoses, for example, psychotic conditions, developmental delay or medical or neurological conditions causing weight loss.

Ancestry-matched controls were selected for each AN case set. Both male and female controls were used (Table 1). These were obtained either from existing collaborations, or through genotyping repository (dbGaP) access. Each site obtained ethical approval from the local ethics committee, and all participants provided written informed consent in accordance with the Declaration of Helsinki.

Population prevalence of AN in these populations ranged from 0.4 to 3% (refs 12, 13, 14, 15, 16, 17, 18; Table 1).

### Genotyping

Cases were genotyped on either the ‘Infinium HumanCoreExome-12 BeadChip Kit (Illumina, San Diego, CA, USA),^19^ or the ‘Infinium HumanCoreExome-24 BeadChip Kit (Illumina),^20^ at the Wellcome Trust Sanger Institute. Where possible, controls were selected from existing studies with matching genotyping platforms to cases. Three control cohorts had been genotyped on the ‘Infinium HumanExome-12 BeadChip Kit’ (Table 1). To ameliorate potential confounding due to chip effects,^21^ chip-type quality control (QC) was carried out, and ~14 000 single-nucleotide polymorphisms (SNPs) removed.

### Quality control

Genotypes were called using the GenCall^22^ and Zcall^23^ algorithms. At each of these genotype-calling stages, QC was performed for each population and for cases and controls separately (Supplementary Table 1). The final number of SNPs included in the analyses is given in Table 2.

### Controlling for population stratification

In order to account for population stratification, a principal components analysis was carried out for each cohort separately using the smartpca software.^24^

Population outliers were identified by merging each population with central European 1000 Genomes data.^25^

Variance explained by each PC was plotted for each population. In order to be both conservative and consistent across populations, the first 10 principal components were included as covariates in the association testing.

### Association testing

Unbalanced case–control ratios can lead to anticonservative *P*-value estimates.^26^ This study includes a number of unbalanced strata (Table 1). The likelihood ratio test has been shown to have low type-I error rate across both balanced and unbalanced cohorts,^26^ and was chosen as the association test for this study.

A lower cutoff of minor allele count of 5 and MAF of 0.1% was used. Association testing was performed for each cohort separately using SNPtest.^27^ In the cohorts with mixed sex controls (all except Italy and Norway), sex was also included as a covariate.

The standard genome-wide significance threshold of *P*⩽5 × 10^−8^ was applied.

### Meta-analysis

Summary statistics across cohort were meta-analyzed using an inverse variance-based test in METAL.^28^ In order to test the heterogeneity of the results, Cochran’s *Q* and the *I*^2^ statistic were computed.

### Assigning variants to genes

Variants identified associated at *P*⩽1 × 10^−4^ were assigned to genes using Ensembl (release 83; Ensembl Genome Browser).^29, 30^ For each variant, all predicted consequences (for example, missense, non-synonymous, and so on) and associated gene transcripts were downloaded and compared. Each variant was associated with only one predicted consequence and one Ensembl gene ID (Ensembl Genome Browser).^29^

### Cluster plot checking

Cluster plots were created for all SNPs reaching *P*⩽1 × 10^−4^ in any analysis (cohort-specific or meta-analysis) using ScatterShot.^31^ SNPs were visually inspected for each cohort, and for cases and controls separately. In instances where multiple cohorts were merged (for example, UK cases), cluster plots were checked separately for each original cohort.

### Burden testing

The potential aggregation of rare variants in cases compared with controls was investigated using a gene-based approach. Burden tests were carried out using the Zeggini–Morris burden test^32^ as implemented in rvtests (Rvtests - Genome Analysis Wiki).

All SNPs with MAF between 0.1 and 5% were included; similar to the single-point analysis, a lower bound of minor allele count=5 was used. A list of genes and locations was obtained from the UCSC genome browser (Table Browser: www.genome.ucsc.edu). All genes with at least two qualifying variants in at least two populations were used, resulting in a total of 9083 genes.

Burden tests were carried out for each population individually, and the results meta-analyzed using Stouffer’s method, weighted according to effective sample size.^33^

The genome-wide significance threshold for burden testing is computed in a similar manner to that for single-point analysis, using Bonferroni correction for the number of genes tested. This results in a genome-wide significance threshold of 5.5 × 10^−6^.

### Pathway analysis

One of the key motivations of studying complex psychiatric disorders such as AN is the desire to unearth biological pathways underlying disease development. Pathway analysis was performed using summary statistics from the meta-analysis for the full data set.

Four pathway databases were used: the Kyoto Encyclopedia of Genes and Genomes (KEGG),^34, 35^ the Reactome pathway database (REACTOME),^36^ PANTHER pathway (PANTHER)^37, 38^ and the Gene Ontology database (GO).^39, 40^ These were curated to remove redundancy, resulting in a total set of 1836 pathways.

The analysis was run once on a merged set of 235 KEGG,^34, 35^ REACTOME^36^ and PANTHER^37, 38^ pathways, and once for the 1601 GO pathways.^39, 40^

Pathway analysis was carried out using MAGMA.^41^ MAGMA was selected for its ability to deal robustly with linkage disequilibrium (LD) between markers, correct for gene length and deal accurately with rare variants. To our knowledge, MAGMA was first used to annotate SNPs to genes. This analysis was repeated twice. In the first analysis, variants were assigned only to the gene they were in, resulting in 68.73% of the variants being assigned to 13 400 genes. In the second analysis, variants were assigned allowing a 20 kb window in both directions from the gene. This procedure included 75.44% of variants across 18 118 genes.

SNP *P*-values were used to create gene scores. The European panel of the 1000 Genomes project was used as a reference set to estimate LD between SNPs. The analysis also requires the sample size of the study to be specified; because of the unbalanced nature of the study, the effective sample sizes were given here.

Gene *P*-values were calculated using MAGMA.^41^ The top 10% of SNPs per gene were used. Significance was defined using a false discovery rate of 5%.^42^

There is a risk when assigning SNPs to genes using MAGMA that some highly associated SNP might be assigned to multiple overlapping genes, and thus distort pathway results. SNP–gene assignments were checked for all pathways that reached false discovery rate-corrected significance. No instances of SNPs being assigned to multiple genes were found across these pathways.

### Replication

SNPs reaching *P*<1 × 10^−4^ in the discovery stage were prioritized for replication. In total, 16 SNPs were selected.

Replication was carried out using two data sets: one existing *in silico* data set and one set for *de novo* genotyping. The *in silico* data set came from an existing GWAS of AN,^7^ genotyped on the Illumina HumanHap610 platform. This data set included 1033 cases and 3733 controls. All cases included in this study were female. Controls were both male and female. The *de novo* replication cohort consisted of 266 self-volunteered female UK cases, collected through the charity Charlotte’s Helix (www.charlotteshelix.net). All participants were adults and had been diagnosed with AN by their clinician. In addition, all participants completed an online questionnaire based on the Structured Clinical Interview^43^ for the Diagnostic and Statistical Manual of Mental Disorders-IV Module H. The Structured Clinical Interview has been used extensively in epidemiological investigations. The Structured Clinical Interview eating disorder module was modified to capture information on lifetime history of eating disorders including AN, and includes questions on body mass index, age of onset, and experience of eating disorders. DNA from the saliva samples was extracted using standard protocols and was quantified using pico-green. Samples were genotyped on the Infinium HumanExome 12 Beadchip, genotypes were called using GenCall and Zcall algorithms and stringent QC was performed pre- and post-call. In all, 1500 ancestry-matched controls (55% female) were obtained from the UK Household Longitudinal Study.

*De novo* genotyping was performed using the iPLEX Assay and the MassARRAY System (Agena Bioscience, San Diego, CA, USA) (formerly Sequenom). Sample and SNP QC were carried out within each replication data set, using an 80% sample call rate and a 90% SNP call rate threshold, and a Hardy–Weinberg equilibrium threshold of 10^−4^. Five samples and one SNP were removed using these criteria.

Post-QC, 15 SNPs and 261 *de novo* cases remained. The *de novo* replication analysis therefore included 15 SNPs, 261 cases and 1500 controls. Genotypes for 12/16 SNPs were available in the *in silico* replication cohort, across 1033 *in silico* cases and 3733 controls.

### Expression analysis

Gene expression data were obtained from the Genotype-Tissue Expression (GTex project) web portal, data release version 6 (dbGap Accession phs000424.v6.p1).^44, 45, 46^

### Power

The sample sizes used in this study are small in the context of other psychiatric phenotypes. Power to identify genome-wide significant signals was calculated using Quanto.^47, 48^ This study is adequately powered to detect low-frequency alleles with large effect sizes and common alleles with substantial effect sizes (80% power to detect common alleles with odds ratio (OR)>1.5; low-frequency alleles with OR>2, Supplementary Figure 1).

### Data availability

Genotypes of European cases included in this study are publicly available through the European Genome-Phenome Archive (EGA), under accession number EGAS00001000913, data set EGAD00010001043, with the exception of German and Dutch genotypes. Genotypes for cases from the United States of America may be obtained through dbGaP. Summary statistics are available for download from the PGC website (https://www.med.unc.edu/pgc/results-and-downloads).

## Results

### GWAS and replication meta-analyses

**Figure 2:**
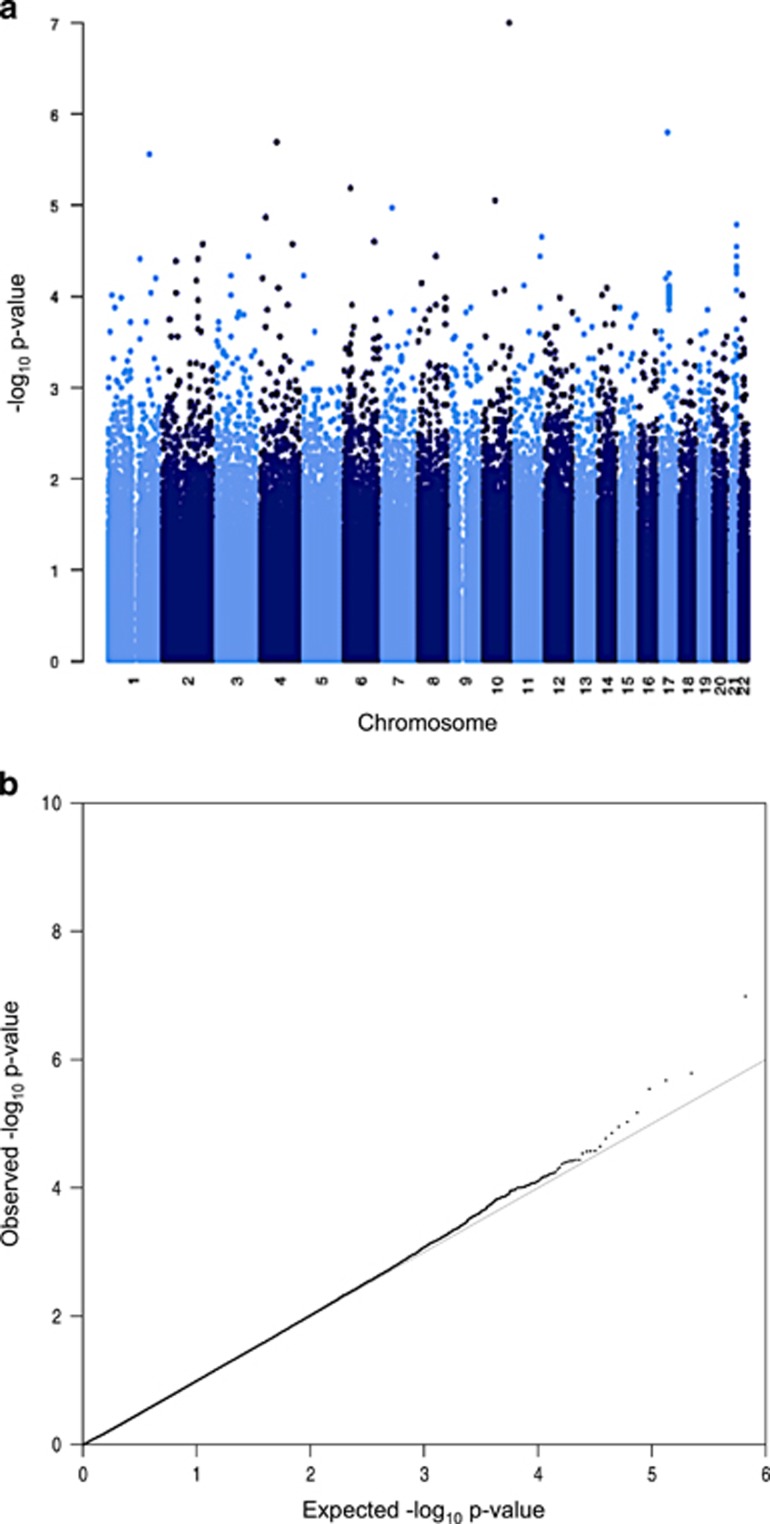
Results from discovery-phase meta-analyses. (**a**) Manhattan plot for meta-analyzed *P*-values, across all nine populations. (**b**) QQ plot (*λ*=0.94).

Association testing was performed separately for each of the nine discovery cohorts within this study (2158 cases, 15 485 controls), and the results were meta-analyzed. No inflation was seen in the QQ plot (Figure 2b). Six variants were identified with *P*<1 × 10^−5^, and nine additional variants with *P*<1 × 10^−4^ (Figure 2a and Supplementary Table 5). Of these, one variant approached genome-wide significance (exm860538/rs199965409, *P*=9.97 × 10^−8^), although this variant is polymorphic only in the Finnish population within these data sets, in the Exome Aggregation Consortium^49^ and in the 1000 Genomes project panel data.^25^ Variants with *P*<1 × 10^−4^ were taken forward for replication.

In total, 16 independent variants were selected for follow-up in one *in silico* cohort (1033 cases, 3733 controls) and one *de novo* genotyping cohort (261 cases, 15 000 controls). Of these, five were low frequency (MAF ~1%) and 11 were common frequency variants.

Twelve signals passed QC and were polymorphic in the *de novo* genotyping cohort, of which four were nominally significant (Supplementary Table 6; *P*<0.05, minimum *P*=0.001). Eight of twelve SNPs had the same direction of effect as in the discovery GWAS, including three of the four nominally significant variants.

Ten of the sixteen variants were present in the *in silico* cohort, of which six had the same direction of effect as in the discovery cohort, and one of these six was associated with *P*=0.02 (Supplementary Table 7).

On the basis of the number of SNPs taken forward for replication, we would not expect to see any variants reaching *P*<0.05 by chance. We also see a higher concordance in direction of effect between discovery and replication cohorts (7/10 in the *in silico* analysis, 8/12 in the *de novo* analysis) than might be expected by chance; however, the number of SNPs tested was too small to achieve statistical significance (*P*=0.17, *P*=0.19, one-sided binomial test).

Five SNPs had the same direction of effect across the meta-analyzed discovery cohort and both replication cohorts. No SNPs reached genome-wide significance in the final global meta-analysis. Two variants were associated with the same direction of effect across discovery and replication cohorts, and reached *P*<0.05 in at least one replication cohort (Table 3).

**Figure 3:**
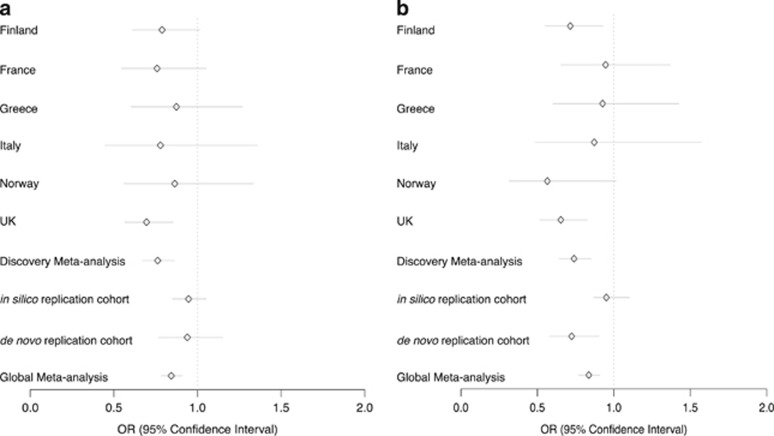
Odds ratios for two notable single-nucleotide polymorphisms (SNPs) across discovery and replication cohorts. (**a**) rs10791286 and (**b**) rs7700147.

rs10791286 was associated with risk for AN across all discovery and replication cohorts (Figure 3a, global *P*=9.89 × 10^−6^, OR 0.84, 95% confidence interval 0.78–0.91). It resides in intron one of the opioid-binding protein/cell adhesion molecule-like (*OPCML*) gene. Data from the CommonMind Consortium project indicate that this variant is an eQTL for *OPCML* in the dorsolateral prefrontal cortex, and is associated with reduced expression (*P*=0.014 after correction for multiple testing).^50^ *OPCML* has a role in opioid-binding and opioid receptor function^51, 52^ and is expressed in a range of neuronal tissues, primarily the cerebellum and cerebellar hemispheres.^44, 45, 46^ *OPCML* has previous associations with body mass index,^53^ waist–hip ratio,^54^ visceral fat distribution^55^ and alcohol dependence,^56^ among other phenotypes. The variant itself has no previously reported associations in any phenotype.

rs7700147 was associated with AN across all discovery and replication cohorts (global *P*=2.93 × 10^−5^, OR 1.2, 95% confidence interval: 1.1, 1.3; Figure 3b). It is an intergenic variant and has no previous associations.

### Burden testing

Burden testing allows the contribution of multiple low-frequency variants to be aggregated across discrete units (for example, genes). Three genes were identified with *P*<1 × 10^−4^, although none reached genome-wide significance (Table 4). A further five genes reached *P*<1 × 10^−4^, but passed inclusion thresholds in one population only (Table 4), and as such are likely to be false-positives.

*FAM96A* has previously been associated with low-density lipoprotein levels and cholesterol^57^ and is primarily expressed in the liver, lymphocytes and adrenal gland.^44, 45, 46^ *KIF7* has no previous phenotype associations and has generally low expression across a wide range of tissues.^44, 45, 46^ *C6orf10* has previous associations with visceral fat^55^ and childhood obesity,^58^ as well as a number of autoimmune disorders.^59, 60, 61, 62, 63, 64^ *C6orf10* is expressed in testes^44, 45, 46^ (see Discussion).

### Biological pathways associated with AN

Allowing a 20 kb window for SNP to gene assignment identified two pathways significant at *q*<0.05: ‘Phospholipase activator’ and ‘GTP-rho binding’ (Table 5).

Using the strictest assignment method of SNPs to genes for the full data set, no pathways were significant after multiple-testing correction. The highest ranking pathway was ‘Calcium ion import’ (*q*-value=0.069).

## Discussion

To our knowledge, this work constitutes the first examination of low frequency (<1% MAF) and rare exonic variation in AN in the context of a genome-wide scan. No low frequency or rare variant replicating associations were identified, although this study was well-powered to detect low-frequency variants with large effect sizes (Supplementary Figure 1). Although polymorphic only in the Finnish population, rs199965409 approached genome-wide significance. It is a non-synonymous variant with a MAF of 0.5% in the Finnish population.^65, 66^ The variant is within the *WDR11* gene, which is associated with hypogonadotropic hypogonadism 14 with or without anosmia.^67, 68, 69^ The clinical features of the disease, such as delayed sexual maturation,^68, 70, 71^ suggest that it may be misdiagnosed or comorbid with AN, which may explain its association in the analysis.

Two notable, but common-frequency, signals were identified with consistent direction of effect across discovery and replication cohorts (rs10791286 and rs7700147). These variants had been removed from the first Genetic Consortium for AN, as part of the Wellcome Trust Case Control Consortium 3 (WTCCC3) AN GWAS because of poor cluster plots; therefore, we were not able to compare effect sizes between studies. Burden tests to investigate an aggregation of rare variants within genes rendered three potentially interesting genes, which require further replication.

Studying rare variation presents a range of challenges. The sample sizes required to identify rare variants with modest effect sizes are substantially larger than for common variants. Further, the MAF spectra seen across *trans*-European populations differ more for rare variants than for common variants, especially when considering genetically distant populations such as Finland and Italy.^25^ This can reduce the power to detect a signal and achieve replication. There are also many technical challenges to consider when conducting a rare variant study; for example, the inflation seen in association tests at low minor allele count^26^ and the increased error rate of calling algorithms when applied to rare variants^22, 23, 72^ We mitigated against the latter challenge by comprehensively examining cluster plots of >10 000 variants that surpassed a *P*-value threshold of *P*<1 × 10^−4^ in any analysis.

Of the genes potentially implicated through the single-point and burden test analyses, three have associations with metabolic and anthropometric phenotypes *(OPCML, C6orf10* and *FAM96a). OPCML* has previously been associated with waist-to-hip ratio, while *C6orf10* has associations with childhood obesity.^58^ *FAM96A* has been shown to be associated with metabolic phenotypes such as low-density lipoprotein and cholesterol levels. The associations of these three genes with metabolic and obesity-related phenotypes may indicate some roles for metabolic processes in AN development, although pathway analysis did not corroborate this observation. A growing body of evidence suggests involvement of metabolic processes in AN development, including appetite-satiety pathways, gut motility and gastric-emptying times.^73, 74, 75, 76, 77, 78, 79^ For example, application of the LD Score regression method revealed significant negative genetic correlations between AN and body mass index, insulin, glucose, and lipid phenotypes and significant positive genetic correlations between AN and HDL cholesterol phenotypes.^1, 80^

Notably, *C6orf10* has been previously associated with childhood obesity.^58^ This finding is particularly interesting for a number of reasons. First, appetite and satiety dysregulation have been shown to be central to the development of childhood obesity.^81, 82^ In particular, reduced satiety responsiveness (experiencing an urge to eat despite internal ‘full’ signals) and heightened responsiveness to food have a role in increased adiposity. Aberrant responses to satiety signals and reduced responsiveness to food are also operative in AN, suggesting shared biological dysregulation between the two conditions.^83, 84^ Children with increased adiposity are at higher risk of eating disorders^85^ as they are more likely to engage in high-risk behaviors such as repeated and excessive dieting and erratic or overly rigid eating patterns.^85, 86, 87, 88, 89^ These children are also at higher risk of being bullied about their weight, which may increase weight and shape concerns, body dissatisfaction and a host of related risk factors for AN development.^85, 86, 87, 88, 89, 90, 91, 92^

The most significant pathway analysis association was with phospholipase activator pathways, which act to catalyze the hydrolysis of glycerophospholipids (GO:0016004 phospholipase activator activity). Phospholipase has a central role in the serotonin-triggered metabolism of arachidonic acid in the brain,^93, 94, 95^ which is a common target for antidepressants^94, 95^ such as lithium, carbamazepine (Tegretol), valproate and lamotrigine (Lamictal).^96^ These antidepressants have been shown to have varying efficacy in treating AN.^97, 98, 99^ Lithium has been used in treatment of AN (with varying success),^97, 98, 99^ while carbamazepine and valproate have been successfully used in individuals with complex comorbid eating disorder phenotypes.^100, 101, 102, 103, 104^ Finally, lamotrigine has been shown to significantly improve eating disorder and mood symptoms in individuals with binge-eating and purging behaviors.^105^

The second pathway identified as significantly associated with AN was GTP-rho binding. This pathway has a role in brain development, and is regulated by autism-susceptibility candidate gene 2 (*AUTS2*).^106^ This finding is consistent with the comorbidity between AN and autism.^107^ Moreover, individuals with AN may be socially withdrawn^107^ and exhibit elevated levels of autistic traits associated with lower social functioning.^107, 108, 109^ *AUTS2* has also been well studied as a candidate gene for alcohol abuse,^110^ which is commonly comorbid with eating disorders.^111^ There is also a well-established link between GTP-rho activation and cognition.^112^ Mice with altered expression of genes regulating Rho-GTPases have been shown to have altered exploratory and anxiety-related behavior, decreased sociability and memory formation, and decreased body weight, among others.^112^ These findings are in line with some of the comorbidities and intermediate phenotypes noted in AN, for example, the high comorbidity with anxiety-related disorders.^113^

There is substantial evidence for the involvement of chromatin-modulating genes in the development of autism,^114, 115, 116, 117, 118, 119^ schizophrenia^120, 121, 122, 123, 124^ and body mass index changes.^114^ Given the comorbidity of these disorders with AN, and the potential overlap with autism indicated in the pathway analysis results, we tested for enrichment of chromatin-modulating genes in these results. We obtained a list of 340 genes involved in modifying chromatin accessibility and/or modifying histone marks from existing literature; of these, 30 reached nominal significance in our burden test, substantially more than expected by chance (binomial test, *P*=0.0026). Moreover, one of the variants identified in the global meta-analysis (exm540361) lays near a gene included in this list (*UHRF1BP1*). Together, these results may indicate a role for chromatin-modifying genes in AN, although more work will be needed to investigate this further.

A number of limitations should be borne in mind when evaluating these results. First, the sample size of this study is small. Psychiatric disorders in general require very large sample sizes in order to identify reliable genome-wide significant signals.^125^ The current study was powered to detect common variants with substantial OR, and rare variants conferring substantial increases in disease risk (OR>2). To our knowledge, this was the first time a study has specifically investigated the role of rare variation in AN, and the lack of low-frequency replicating findings may indicate that little advancements may be made in this particular genomic search space.

We did not see any overlap between the pathways identified here and those identified in the recent PGC pathway analysis;^126^ however, this may reflect the relatively small sample size of this study, as well as different pathway analysis methodologies used.

In this study we only examined female AN cases of European origin. It has been suggested that the genetics underlying AN development may be easier to assess in an all-male study,^3^ as there may be a greater genetic risk required to induce trait expression. The higher relative risk in male subjects may also reflect this.^3^ To date, this has not been possible because of the lower prevalence of the disorder in men, resulting in substantially smaller sample sizes. Moreover, if AN is heterogeneous between populations, in order to fully understand the genetic etiology of the disorder, it will be necessary to expand collection to include more diverse samples. Efforts are already underway in a number of Asian populations such as Taiwan, Japan, Korea and China, as well as some South American populations such as Argentina and Brazil.

A caveat to this study is that controls were not screened for AN, and that both male and female controls were used. Given the population prevalence of AN across population of European descent, ~80 female and ~10 male controls would be expected to have AN diagnoses. Given the low rate of treatment seeking in AN,^127^ it would not be possible to confidently screen population-based or previously existing control cohorts for AN.

The underlying biological etiology of AN is complex and has not been elucidated yet. Here we have identified a number of variants that warrant follow-up in larger sample sizes, and which point to a role for metabolic, appetite-related and obesity-related effects, in line with a growing body of evidence for metabolic involvement in AN development. Substantially increased sample sizes and detailed phenotyping to reduce heterogeneity will be necessary to empower the characterization of the genetic architecture of AN.

## Figures and Tables

**Table 1:** Final numbers of cases and controls after QC

*Population (abbreviation)*	*Cases*	*Controls (% female)*	*Control source*	*AN population prevalence*
Germany (DE)	664	2701 (51.8)	Pre-existing study	0.93% (11)
Finland (FIN)	136	5082 (54.7)	Pre-existing study	2.2% (12)
France (FR)	215	208 (75.5)	Pre-existing study	0.93% (11)
Greece (GR)	78	408 (58.1)	In-house	—
Italy (ITA)	103	48 (100)	In-house	1.3% (13)
Netherlands (NL)	290	3071 (49.7)	In-house; pre-existing study	1.3% (14)
Norway (NO)	80	94 (100)	In-house	3.0% (15)
UK	217	3000 (56.7)	Pre-existing study^a^	0.64% (16)
USA	375	873 (50.6)	Obtained from dbGaP	0.9% (17)
Total	2158	15 485		

Abbreviations: AN, anorexia nervosa; QC, quality control.

a In all, 3000 UK controls were randomly selected from the 9828 samples, genotyped as part of the UK household longitudinal survey. The UK Household Longitudinal Study is led by the Institute for Social and Economic Research at the University of Essex and funded by the Economic and Social Research Council. The survey was conducted by NatCen and the genome-wide scan data were analyzed and deposited by the Wellcome Trust Sanger Institute. Information on how to access the data can be found on the Understanding Society website https://www.understandingsociety.ac.uk/.

**Table 2:** Final number of SNPs per population

*Population*	*Final number of SNPs*
DE	234 736
FR	524 954
FIN	524 786
GR	517 910
ITA	522 430
NL	229 136
NO	513 082
UK	510 200
USA	235 975

Abbreviations: DE, Germany; FIN, Finland; FR, France; GR, Greece; ITA, Italy; NL, Netherlands; NO, Norway; SNP, single-nucleotide polymorphism.

**Table 3:** Global meta-analysis results

*Chr*	*Pos*	*Id*	*Associated gene*	*EA*	*NEA*	*EAF*	*OR*	*OR_95L*	*OR_95U*	P	*Het_chisq*	*N_st (discovery/replication)*
2	195032811	kgp3754622 (rs75245228)	*—*	a	g	0.052	0.81	0.69	0.96	0.016	12.739	8 (6/2)
11	133096498	rs10791286	*OPCML*	a	g	0.33	0.84	0.78	0.91	9.40 × 10^−6^	4.228	8 (6/2)
10	53754335	rs1904050	*PRKG1*	a	g	0.20	0.87	0.79	0.95	0.0018	15.033	7 (5/2)
11	125655014	rs536968	*PATE3*	a	g	0.12	0.88	0.79	0.98	0.023	12.082	8 (6/2)
10	122659625	exm860538 (rs199965409)	*WDR11*	a	g	<0.01	10.42	4.40	24.69	9.97 × 10^−8^	0	1 (1/0)
4	157167891	rs7700147	***ANKRD50***	t	c	0.21	1.20	1.10	1.30	2.79 × 10^−5^	9.093	8 (6/2)
6	34826040	exm540361 (rs200155060)	*UHRF1BP1*	a	g	<0.01	0.18	0.08	0.37	6.47 × 10^−6^	0	1 (1/0)
6	147840595	rs669830	*SAMD5*	t	g	0.26	1.11	1.01	1.21	0.029	13.174	5 (3/2)
21	47963149	rs11701571	*DIP2A*	a	g	0.24	1.11	1.02	1.21	0.011	12.185	6 (4/2)
7	49620107	rs10264162	***VWC2***	t	g	0.43	0.91	0.84	0.97	0.0068	23.105	8 (6/2)
1	197404688	exm134618 (rs142090517)	*CRB1*	a	g	<0.01	11.97	4.24	33.81	2.76 × 10^−6^	0	1 (1/0)
3	150748151	rs1703802	***CLRN1-AS1***	t	g	0.12	0.84	0.75	0.93	0.00085	7.007	8 (6/2)
17	31082572	exm1310689 (rs145290255)	*MYO1D*	t	c	0.0011	0.02	0.00	0.10	1.74 × 10^−6^	0.276	2 (1/1)
4	80949829	exm-rs4333130	*ANTRX2*	t	c	0.38	0.89	0.83	0.94	7.14 × 10^−5^	7.526	10 (8/2)
4	26482021	rs2854030	*CCKAR*	t	c	0.31	0.88	0.78	0.98	0.021	23.181	8 (6/2)

Abbreviations: CHR, chromosome; EA, effect allele; EAF, effect allele frequency; *I*_2_, measure of heterogeneity; NEA, non-effect allele; N_st, number of contributing studies; OR, odds ratio; OR_ 95L, lower 95% confidence interval; OR_95U, upper 95% confidence interval; P, *P*-value; POS, position in hg18.

Gene names given are best-redicted consequence from ensembl,^24,25^ where none is available; the nearest gene is given instead in bold.

**Table 4:** Burden test results

*Genes*	P*-value*	*Populations*
*KIF7*	7.85 × 10^−5^	DE, FIN, NL, UK, USA
*FAM96A*	6.82 × 10^−5^	GR, UK
*C6orf10*	8.32 × 10^−5^	DE, FIN, FR, GR, ITA, NL, NO, UK USA
*ATP2C1*	6.03 × 10^−9^	ITA
*SPINK6*	6.03 × 10^−9^	ITA
*RP11-550C4.6*	6.03 × 10^−9^	ITA
*C15orf57*	6.03 × 10^−9^	ITA
*C11orf68*	1.75 × 10^−10^	NL

Abbreviations: DE, Germany; FIN, Finland; FR, France; GR, Greece; ITA, Italy; NL, Netherlands; NO, Norway.

**Table 5:** Pathway analysis results for full data set

*Window*	*Pathway*	*Identifier*	P*-value*	q*-value*
±20 kb	Phospholipase activator	GO:0016004	6.6 × 10^−6^	0.011
	GTP-rho binding	GO:0017049	1.9 × 10^−5^	0.03
0	Calcium ion import	GO:0070509	4.3 × 10^−5^	0.069

Abbreviation: GO, Gene Ontology database.
