# Supplementary material for: Investigation of common, low-frequency and rare genome-wide variation in anorexia nervosa
Source: Mol Psychiatry. 2017 Jul 25;23(5):1169–80. doi: 10.1038/mp.2017.88 (PMC5828108; doi:10.1038/mp.2017.88)
Supplement: Supplementary Table 1 [file mp201788x1.docx]

**Suppl. Table 1: Number of cases at each QC stage**. Genotypes were first called using gen-call, and QC carried out (QC-stage 1). Genotypes were then re-called using z-call, and QC repeated (QC stage 2).

| **Population** | **Original number of cases** | **Number of failed cases** | | **Final number of cases** |
| --- | --- | --- | --- | --- |
|  |  | **Stage 1** | **Stage 2** |  |
| DE | 728 | 47 | 17 | 664 |
| FIN | 163 | 20 | 7 | 136 |
| FR | 237 | 18 | 4 | 215 |
| GR | 89 | 11 | 0 | 78 |
| ITA | 168 | 63 | 2 | 103 |
| NL | 357 | 55 | 12 | 290 |
| NO | 87 | 4 | 3 | 80 |
| UK (CoreExome 12.0) | 181 | 64 | 5 | 112 |
| UK (CoreEXome 24.0) | 166 | 52 | 9 | 105 |
| USA | 497 | 114 | 8 | 375 |
