# Supplementary material for: Investigation of common, low-frequency and rare genome-wide variation in anorexia nervosa
Source: Mol Psychiatry. 2017 Jul 25;23(5):1169–80. doi: 10.1038/mp.2017.88 (PMC5828108; doi:10.1038/mp.2017.88)
Supplement: Supplementary Table 2 [file mp201788x2.docx]

**Suppl. Table 2: Number of controls at each QC stage.**

| **Population** | **Original number of controls** | **Number of failed controls** | | **Final number of controls** |
| --- | --- | --- | --- | --- |
|  |  | **Stage 1** | **Stage 2** |  |
| DE | 2,921 | 169 | 51 | 2,701 |
| FIN | 5,550 | 275 | 193 | 5,082 |
| FR | 320 | 107 | 5 | 208 |
| GR | 436 | 23 | 5 | 408 |
| ITA | 56 | 8 | 0 | 48 |
| NL (CoreExome 12.0) | 268 | 0 | 10 | 258 |
| NL (Exome 12.0) | 3,181 | 259 | 111 | 2,811 |
| NO | 100 | 3 | 3 | 94 |
| UK | 10,484 | 450 | 204 | 9,828 |
| USA | 1,051 | 130 | 48 | 873 |
