# Supplementary material for: Investigation of common, low-frequency and rare genome-wide variation in anorexia nervosa
Source: Mol Psychiatry. 2017 Jul 25;23(5):1169–80. doi: 10.1038/mp.2017.88 (PMC5828108; doi:10.1038/mp.2017.88)
Supplement: Supplementary Table 3 [file mp201788x3.docx]

**Suppl. Table 3: Number of SNPs at each QC stage (cases)**

| **Population** | **Original number of SNPs** | **Number of failing SNPs** | |
| --- | --- | --- | --- |
|  |  | **Stage 1** | **Stage 2** |
| DE | 538,403 | 6,306 | 1,082 |
| FR | 538,403 | 6,804 | 2,241 |
| FIN | 538,403 | 3,864 | 2,215 |
| GR | 538,403 | 6,256 | 6,466 |
| ITA | 547,589 | 6,772 | 4,591 |
| NL | 538,403 | 4,875 | 9,999 |
| NO | 538,403 | 2,400 | 4,878 |
| UK (CoreExome 12.0) | 538,403 | 3,614 | 3,311 |
| UK (CoreExome 24.0) | 547,589 | 3,618 | 2,249 |
| USA | 538,403 | 3,415 | 2,164 |
