# Supplementary material for: Investigation of common, low-frequency and rare genome-wide variation in anorexia nervosa
Source: Mol Psychiatry. 2017 Jul 25;23(5):1169–80. doi: 10.1038/mp.2017.88 (PMC5828108; doi:10.1038/mp.2017.88)
Supplement: Supplementary Table 4 [file mp201788x4.docx]

**Suppl. Table 4: Number of SNPs at each QC stage (controls)**

| **Population** | **Original number of SNPs** | **Number of failing SNPs** | |
| --- | --- | --- | --- |
|  |  | **Stage 1** | **Stage 2** |
| DE | 247,870 | 1,585 | 712 |
| FR | 538,403 | 6,266 | 1,470 |
| FIN | 535,735 | 5,937 | 5,814 |
| GR | 538,403 | 7,618 | 3,423 |
| ITA | 547,589 | 8,194 | 10,184 |
| NL (CoreExome 12.0) | 534,682 | 3,412 | 11,127 |
| NL (Exome 12.0) | 239,525 | 4,712 | 1,308 |
| NO | 538,403 | 3,967 | 17,691 |
| UK | 538,448 | 11,070 | 4,661 |
| USA | 247,475 | 1,075 | 816 |
