# Supplementary material for: Investigation of common, low-frequency and rare genome-wide variation in anorexia nervosa
Source: Mol Psychiatry. 2017 Jul 25;23(5):1169–80. doi: 10.1038/mp.2017.88 (PMC5828108; doi:10.1038/mp.2017.88)
Supplement: Supplementary Table 5 [file mp201788x5.docx]

**Suppl. Table 5: Discovery ORs**

| Chr | Pos | Id | Associated gene | EA | NEA | EAF | OR | OR_95L | OR_95U | P |
| --- | --- | --- | --- | --- | --- | --- | --- | --- | --- | --- |
| 2 | 195032811 | kgp3754622 (rs75245228) | *-* | a | g | 0.052 | 0.59 | 0.46 | 0.76 | 2.65x10^-5^ |
| 11 | 133096498 | rs10791286 | *OPCML* | a | g | 0.33 | 0.76 | 0.67 | 0.86 | 2.2 x10^-5^ |
| 10 | 53754335 | rs1904050 | *PRKG1* | a | g | 0.20 | 0.70 | 0.59 | 0.82 | 9.06 x10^-6^ |
| 11 | 125655014 | rs536968 | *PATE3* | a | g | 0.12 | 0.69 | 0.58 | 0.82 | 3.66 x10^-5^ |
| 10 | 122659625 | exm860538 (rs199965409) | *WDR11* | a | g | <0.01 | 10.42 | 4.40 | 24.69 | 9.95 x10^-8^ |
| 4 | 157167891 | rs7700147 | ***ANKRD50*** | t | c | 0.21 | 1.35 | 1.17 | 1.56 | 2.59 x10^-5^ |
| 6 | 34826040 | exm540361 (rs200155060) | *UHRF1BP1* | a | g | <0.01 | 0.18 | 0.08 | 0.37 | 6.47 x10^-6^ |
| 6 | 147840595 | rs669830 | *SAMD5* | t | g | 0.26 | 1.51 | 1.25 | 1.82 | 2.56 x10^-5^ |
| 21 | 47963149 | rs11701571 | *DIP2A* | a | g | 0.24 | 1.37 | 1.19 | 1.58 | 1.64x10^-5^ |
| 7 | 49620107 | rs10264162 | ***VWC2*** | t | g | 0.43 | 0.76 | 0.67 | 0.86 | 1.08 x10^-5^ |
| 1 | 197404688 | exm134618 (rs142090517) | *CRB1* | a | g | <0.01 | 11.97 | 4.24 | 33.81 | 2.76 x10^-6^ |
| 3 | 150748151 | rs1703802 | ***CLRN1-AS1*** | t | g | 0.12 | 0.70 | 0.60 | 0.83 | 3.71 x10^-5^ |
| 17 | 31082572 | exm1310689 (rs145290255) | *MYO1D* | t | c | 0.0011 | 0.02 | 0.00 | 0.10 | 1.59 x10^-6^ |
| 4 | 80949829 | exm-rs4333130 | *ANTRX2* | t | c | 0.38 | 0.82 | 0.76 | 0.89 | 2.05 x10^-5^ |
| 4 | 26482021 | rs2854030 | *CCKAR* | t | c | 0.31 | 0.75 | 0.65 | 0.85 | 1.35 x10^-5^ |

Abbreviations: CHR, chromosome; POS, position in hg18; EA, effect allele; NEA, non-effect allele; EAF, effect allele frequency; OR, odds ratio; OR_

95L, lower 95% confidence interval; OR_95U, upper 95% confidence interval; P, P-value; Gene names given are best predicted consequence from ensembl^24,25^; where none is available, the nearest gene is given instead, in bold.
