# Supplementary material for: Investigation of common, low-frequency and rare genome-wide variation in anorexia nervosa
Source: Mol Psychiatry. 2017 Jul 25;23(5):1169–80. doi: 10.1038/mp.2017.88 (PMC5828108; doi:10.1038/mp.2017.88)
Supplement: Supplementary Table 6 [file mp201788x6.docx]

**Suppl. Table 6: Replication ORs (de-novo replication cohort)**

| Chr | Pos | Id | Associated gene | EA | NEA | OR | OR_95L | OR_95U | P |
| --- | --- | --- | --- | --- | --- | --- | --- | --- | --- |
| 2 | 195032811 | kgp3754622 (rs75245228) | *-* | a | g | 0.98 | 0.60 | 1.60 | 0.92 |
| 11 | 133096498 | rs10791286 | *OPCML* | a | g | 0.94 | 0.77 | 1.15 | 0.54 |
| 10 | 53754335 | rs1904050 | *PRKG1* | a | g | 0.77 | 0.62 | 0.97 | 0.03 |
| 11 | 125655014 | rs536968 | *PATE3* | a | g | 1.04 | 0.77 | 1.41 | 0.79 |
| 10 | 122659625 | exm860538 (rs199965409) * | *WDR11* | a | g | NA | NA | NA | NA |
| 4 | 157167891 | rs7700147 | ***ANKRD50*** | t | c | 1.38 | 1.11 | 1.72 | 0.0051 |
| 6 | 34826040 | exm540361 (rs200155060) * | *UHRF1BP1* | a | g | NA | NA | NA | NA |
| 6 | 147840595 | rs669830 | *SAMD5* | t | g | 1.06 | 0.84 | 1.32 | 0.64 |
| 21 | 47963149 | rs11701571 | *DIP2A* | a | g | 1.01 | 0.81 | 1.26 | 0.94 |
| 7 | 49620107 | rs10264162 | ***VWC2*** | t | g | 1.35 | 1.10 | 1.66 | 0.0032 |
| 1 | 197404688 | exm134618 (rs142090517) * | *CRB1* | a | g | NA | NA | NA | NA |
| 3 | 150748151 | rs1703802 | ***CLRN1-AS1*** | t | g | 1.00 | 0.74 | 1.34 | 1.00 |
| 17 | 31082572 | exm1310689 (rs145290255) | *MYO1D* | t | c | 187.6 | 0.00 | 9.87x10^16^ | 0.28 |
| 4 | 80949829 | exm-rs4333130 | *ANTRX2* | t | c | 0.93 | 0.77 | 1.14 | 0.50 |
| 4 | 26482021 | rs2854030 | *CCKAR* | t | c | 0.70 | 0.56 | 0.88 | 0.0012 |

Abbreviations: CHR, chromosome; POS, position in hg18; EA, effect allele; NEA, non-effect allele; OR, odds ratio; OR_

95L, lower 95% confidence interval; OR_95U, upper 95% confidence interval; P, P-value; Gene names given are best predicted consequence from ensembl^24,25^; where none is available, the nearest gene is given instead, in bold. Variants with the same direction of effect as in the discovery sample are highligjted in green.

*These variants were monomorphic in this cohort.
