# Supplementary material for: Investigation of common, low-frequency and rare genome-wide variation in anorexia nervosa
Source: Mol Psychiatry. 2017 Jul 25;23(5):1169–80. doi: 10.1038/mp.2017.88 (PMC5828108; doi:10.1038/mp.2017.88)
Supplement: Supplementary Table 7 [file mp201788x7.docx]

**Suppl. Table 7: Replication ORs (in-silico replication cohort)**

| Chr | Pos | Id | Associated gene | EA | NEA | OR | OR_95L | OR_95U | P |
| --- | --- | --- | --- | --- | --- | --- | --- | --- | --- |
| 2 | 195032811 | kgp3754622 (rs75245228) | *-* | a | g | 1.12 | 0.86 | 1.46 | 0.39 |
| 11 | 133096498 | rs10791286 | *OPCML* | a | g | 0.88 | 0.79 | 0.98 | 0.02 |
| 10 | 53754335 | rs1904050 | *PRKG1* | a | g | 1.023 | 0.90 | 1.16 | 0.72 |
| 11 | 125655014 | rs536968 | *PATE3* | a | g | 1.02 | 0.88 | 1.18 | 0.81 |
| 10 | 122659625 | exm860538 (rs199965409) * | *WDR11* | a | g | NA | NA | NA | NA |
| 4 | 157167891 | rs7700147 | ***ANKRD50*** | t | c | 1.05 | 0.93 | 1.18 | 0.41 |
| 6 | 34826040 | exm540361 (rs200155060) * | *UHRF1BP1* | a | g | NA | NA | NA | NA |
| 6 | 147840595 | rs669830 | *SAMD5* | t | g | 1.01 | 0.89 | 1.12 | 0.98 |
| 21 | 47963149 | rs11701571 | *DIP2A* | a | g | 0.99 | 0.89 | 1.12 | 0.99 |
| 7 | 49620107 | rs10264162 | ***VWC2*** | t | g | 0.92 | 0.84 | 1.02 | 0.11 |
| 1 | 197404688 | exm134618 (rs142090517) * | *CRB1* | a | g | NA | NA | NA | NA |
| 3 | 150748151 | rs1703802 | ***CLRN1-AS1*** | t | g | 0.92 | 0.80 | 1.07 | 0.28 |
| 17 | 31082572 | exm1310689 (rs145290255) * | *MYO1D* | t | c | NA | NA | NA | NA |
| 4 | 80949829 | exm-rs4333130 | *ANTRX2* | t | c | 0.99 | 0.89 | 1.09 | 0.74 |
| 4 | 26482021 | rs2854030* | *CCKAR* | t | c | NA | NA | NA | NA |

Abbreviations: CHR, chromosome; POS, position in hg18; EA, effect allele; NEA, non-effect allele; OR, odds ratio; OR_

95L, lower 95% confidence interval; OR_95U, upper 95% confidence interval; P, P-value; Gene names given are best predicted consequence from ensembl^24,25^; where none is available, the nearest gene is given instead, in bold. Variants with the same direction of effect as in the discovery sample are highlighted in green.

*These variants were monomorphic or not included in the CHOP analysis summary statistic
