# Supplementary material for: Investigation of common, low-frequency and rare genome-wide variation in anorexia nervosa
Source: Mol Psychiatry. 2017 Jul 25;23(5):1169–80. doi: 10.1038/mp.2017.88 (PMC5828108; doi:10.1038/mp.2017.88)
Supplement: Supplementary Figure 1 [file mp201788x8.docx]

**B**

**A**

**Supplementary Figure 1:** **Power calculations. Lines are shown for 50%, 80% and 99% power.**

1. Power calculated for a range of MAF and OR.
2. Power calculations shown in detail for MAF<0.02
